# Replicative conditioning of *Herpes simplex* type 1 virus by Survivin promoter, combined to ERBB2 retargeting, improves tumour cell-restricted oncolysis

**DOI:** 10.1038/s41598-020-61275-w

**Published:** 2020-03-09

**Authors:** Emanuele Sasso, Guendalina Froechlich, Gabriella Cotugno, Anna Morena D’Alise, Chiara Gentile, Veronica Bignone, Maria De Lucia, Biljana Petrovic, Gabriella Campadelli-Fiume, Elisa Scarselli, Alfredo Nicosia, Nicola Zambrano

**Affiliations:** 1 Dipartimento di Medicina Molecolare e Biotecnologie Mediche, Università degli Studi di Napoli Federico II, Via S. Pansini, 5, 80131 Naples, Italy; 2 CEINGE Biotecnologie Avanzate S.C.aR.L., Via G. Salvatore 486, 80145 Naples, Italy; 3 Nouscom S.R.L., Via di Castel Romano 100, 00128 Rome, Italy; 4 Department of Experimental, Diagnostic and Specialty Medicine, University of Bologna, Via San Giacomo 12, 40126 Bologna, Italy

**Keywords:** Cancer immunotherapy, Targeted therapies, Herpes virus

## Abstract

Oncolytic virotherapy is emerging as a promising therapeutic option for solid tumours. Several oncolytic vectors in clinical testing are based on attenuated viruses; thus, efforts are being taken to develop a new repertoire of oncolytic viruses, based on virulent viral genomes. This possibility, however, raises concerns dealing with the safety features of the virulent phenotypes. We generated a double regulated *Herpes simplex* type-1 virus (HSV-1), in which tumour cell restricted replicative potential was combined to selective entry via ERBB2 receptor retargeting. The transcriptional control of the viral alpha4 gene encoding for the infected cell protein-4 (ICP4) by the cellular Survivin/*BIRC5* promoter conferred a tumour cell-restricted replicative potential to a virulent HSV-1 genome. The combination of the additional ERBB2 retargeting further improved the selectivity for tumour cells, conferring to the double regulated virus a very limited ability to infect and propagate in non-cancerous cells. Accordingly, a suitable replicative and cytotoxic potential was maintained in tumour cell lines, allowing the double regulated virus to synergize *in vivo* with immune checkpoint (anti-PD-1) blockade in immunocompetent mice. Thus, restricting the replicative spectrum and tropism of virulent HSV-1 genomes by combination of conditional replication and retargeting provides an improved safety, does not alter the oncolytic strength, and is exploitable for its therapeutic potential with immune checkpoint blockade in cancer.

## Introduction

Oncolytic viruses (OVs) represent a class of natural or engineered viral species, possessing the ability to infect cancer cells, while sparing healthy tissues^1^. This selectivity is usually conferred by attenuating mutations, resulting in preferential replication of viral genomes in tumour cells^2^. Besides reducing the tumour bulk, OVs also act as immunotherapeutic agents. This feature is principally due to the immunogenic cell death (ICD) mechanisms induced by OVs, including immunogenic apoptosis, necrosis, necroptosis, pyroptosis, and autophagic cell death^3–5^. Viral infection also induces the release of stimulating cytokines, such as IL-1, IL-6, IL-12, IL-18, IFN-γ. Along with these molecules, lysed cancer cells release tumour-associated antigens and cancer-related proteins. These mechanisms allow the immunosuppressed tumour microenvironment (TME) to turn into an immunocompetent habitat. This effect is potentiated in OVs, in which immunostimulatory cytokines or chemokines are encoded by engineered viral genomes, and can synergize with immune checkpoint blockade^6–8^. This may translate into a therapeutic opportunity to potentiate the effects of immune checkpoint blockade in patients refractory to immunotherapy^9,10^. Talimogene laherparepvec (T-VEC), a *Herpes simplex*-based OV (oHSV), was approved by FDA in 2015 for treatment of recurrent melanoma after initial surgery^11,12^. It holds a *ICP34.5*-deleted genome, resulting in attenuated neurovirulence, and diminished infection of normal cells. Transgenic expression of GM-CSF represents an additional feature of T-VEC, conferring the ability to activate antigen-presenting cells (APC) within the tumour microenvironment^13^. Despite these features, the need for a repertoire of viral vectors with improved properties stimulated the development of new strategies for oncolytic virotherapy, taking into account both safety and potency of oncolytic viruses. Different strategies have been devised in order to maintain selectivity of infection for cancer cells, while keeping an effective replicative and lytic potential of OVs. Among these, engineered oHSV with specific tropism for cancer cells have been generated on virulent, non-attenuated genomes^14–16^. Transcriptional retargeting is based on substitution of endogenous promoters, controlling the expression of viral genes, such as *ICP4* and *ICP27*, with cellular promoters which confer tumour-restricted expression and lead to viral selective replication in cancer cells^17–22^. The insertion of sequences within relevant HSV-1 genes, targeted by miRNAs selectively expressed in normal cells, represents an additional strategy for limiting off-tumour replication of virulent oHSVs^23^. Combination of strategies, such as transcription and translation retargeting, have also been exploited, in order to obtain tumour-specific oncolytic viruses^24,25^.

Receptor retargeting of HSV-1 is a prominent feature for tumour-restricted tropism of oHSVs. Several tumours, indeed, express selected cell surface receptors, which can be recognized by engineered viral glycoproteins fused to antibody fragments or receptor ligands. ERBB2 retargeted viruses have been extensively characterized for the specific tropism for ERBB2 positive tumours^14,15^; fully virulent oHSV with restricted tropism to ERBB2 expressing tumours proved to be efficacious in preclinical models^26^. Very recently, a ERBB2-retargeted oHSV armed with IL-12 proved to be efficacious in subcutaneous and glioblastoma tumour models^27,28^. Accordingly, combination of OVs and immune checkpoint inhibitors have shown improved clinical activity^8,29,30^.

In this paper, we describe the implementation and the characterization of a non-attenuated, virulent oHSV obtained by combining cancer selective replication by transcription control and receptor retargeting. The characterization of tumour associated promoters highlighted the suitability of the Survivin promoter for restriction of viral replication in tumour cell lines by conditional expression of the immediate-early *ICP4* viral gene. ERBB2 receptor retargeting was finally combined to the tumour cell-restricted replication feature for selective infection of ERBB2-positive cells. The resulting, double regulated oHSV showed improved specificity for cancer cells as compared to non-cancerous ones, and comparable *in vitro* oncolytic activity to the targeted virus. The double regulated oHSV also showed unaltered oncolytic potential *in vivo* compared to the retargeted virus in a combination therapy setting of oncolytic virotherapy with PD-1 checkpoint blockade. Thus, our data show that the added feature of cancer cell-restricted replicative potential to receptor retargeting may actually improve the safety feature of oncolytic virotherapy.

## Materials and Methods

### Cell lines and reporter assays

SKOV3 and SAN cell lines were cultured in RPMI Medium 1640-GlutaMAX™-I; HEK293, A375 and LLC1-ERBB2 cells were cultured in Dulbecco’s Modified Eagle’s Medium; MRC5 cells were cultured in Minimum Essential Medium Eagle. All media were supplemented with 10% heat-inactivated foetal bovine serum (FBS), 50 UI ml^−1^ penicillin, 50 µg ml^−1^ streptomycin, 2 mM L-glutamine. LLC1-ERBB2 medium was supplemented with puromycin to maintain stable expression of human ERBB2 transgene. All the reagents for cell culturing were from Gibco^TM^, Thermo Fisher Scientific. Cell lines were purchased from the American Type Culture Collection (ATCC) or kindly donated from collaborators and cultured in a humidified atmosphere containing 5% CO_2_ at 37 °C.

The putative promoter sequences for *Survivin*, *TERT* and *CXCR4* genes were synthesized by The Invitrogen GeneArt Gene Synthesis service and were subcloned into pSEAP2-Basic vector (GenBank Accession#: U89937, Clontech Laboratories, Mountain View, CA, USA) upstream SEAP cDNA by *Xho*I and *Hin*dIII restriction enzymes. HEK293, SKOV3, A375, SAN and MRC5 cells were transfected with the specific promoter-SEAP vectors by using Lipofectamine 2000 Transfection Reagent (Life Technologies, Inc.) and grown up for 24 hours. SEAP activity was dosed from conditioned media by Phospha-Light SEAP Reporter Gene Assay System (Thermo Fisher). Assays were performed according to the manufacturer’s instructions in 96-well plates. For measurement of *Survivin* promoter-SEAP in response to Nocodazole, SKOV3 cells were transfected with *Survivin* promoter-SEAP vector and 8 h after, Nocodazole was added to the media at a final concentration of 0.1 µg/ml. 12 h post Nocodazole treatment, SEAP activity was dosed from conditioned media. Cell lysis following viral infection was assessed by measuring the release of extracellular lactate dehydrogenase (LDH) by Pierce LDH Cytotoxicity Assay Kit (Thermofisher Scientific) according to the manufacturer’s recommendations.

### Modifications of BAC-HSV-1 vectors

We used the *sacB*/*amp*^R^/*lac*Z recombineering system to modify HSV-1 vectors. Briefly, a positive selection step followed by a negative one were necessary to engineer BAC recombinant R-LM55 derived from wild type strain F HSV-1 (GenBank accession number: GU734771.1) with BAC insertion in UL3-UL4 intergenic region. For the generation of Survivin_oHSV and SurE_oHSV, in the first step, a DNA fragment containing the *sacB*/*amp*^R^/*lac*Z selection cassette was amplified by PCR from a donor plasmid with Phusion Hot Start II High-Fidelity DNA Polymerase (Thermo Scientific) in a 50 µl volume in 1X Phusion HF containing 200 µM dNTPs, 3% DMSO (v/v), 0.5 µM primers, 0.02 U/µL Phusion Hot Start II DNA Polymerase and 10 ng of template. The oligonucleotides used for this amplification contained in their 5′-end at least 40 base-pairs of perfect homology to the region to be engineered (*ICP4* promoter or *gD* gene). The PCR products were purified from 1% agarose gel with Wizard® SV Gel and PCR Clean-Up System (Promega). The cassettes were electroporated (25 mF, 2.5 kV, 200 Ohm) into electrocompetent SW102 heat-induced bacteria containing the BAC-HSV-1 (R-LM55) of interest. After 1 h recovery, SW102 cells were plated on LB agar plus 12.5 µg/ml chloramphenicol, 20 μg/ml ampicillin, 80 μg/ml X-gal and 200 μM IPTG. The blue colonies were cultured in LB medium for 16 hours, and DNA was extracted by NucleoBond PC100 (MACHEREY-NAGEL GmbH & Co. KG). The second step of recombineering was performed by transformation by electroporation of SW102 cells, derived from the first selection step, with the DNA fragment containing the Survivin promoter or the anti-ERBB2 antibody fragment scFv amplified with 40 base-pair extensions for perfect homology to the region of interest within the HSV-1 genome. The negative selection was performed on plates containing sucrose. Since *ICP4* is present in two copies, a 19 base pair tag was inserted into the second *locus*, upstream the Survivin promoter, to distinguish from each other. A detailed list of oligonucleotides is reported in Table 1. For *in vivo* studies, the BAC region flanked by *loxP* elements was removed by Cre recombinase in order to avoid immunological interference by BAC encoded elements (e.g. eGFP and chloramphenicol resistance).

**Table 1:** Oligonucleotide sequences.

Step I RC1_Fwd	5′-gcccggggacggccaacgggcgcgcggggctcgtatctcattaccgccgaacccctatttgtttatttttct-3′
Step I RC1_Rev	5′-gcggtcccgcgtcgggtcgtggatccgtgtcggcagccgcgctccgtgtgttatttgttaactgttaattgtc-3′
Step II RC1_Fwd	5′-gcccggggacggccaacgggcgcgcggggctcgtatctcattaccgccgagttctttgaaagcagtcgag-3′
Step II RC1v1_Fwd	5′-cccggggacggccaacgggcgcgcggggctcgtatctcattaccgccgaatatggatcctatggcgcggttctttgaaagcagtcgag-3′
Step II RC1_Rev	5′-gcggtcccgcgtcgggtcgtggatccgtgtcggcagccgcgctccgtgtggccgccgccgccacctct-3′
Step I RC2_Fwd	5′-gggaagtcggggcccgggccccgcccccggcccgttcctcgttagcatgcacccctatttgtttatttttct-3′
Step I RC2_Rev	5′-gccggggcgctgcttgttctccgacgccatcgccgatgcggggcgatcctttatttgttaactgttaattgtc-3′
Step II RC2_Fwd	5′-gggaagtcggggcccgggccccgcccccggcccgttcctcgttagcatgcgttctttgaaagcagtcgag-3′
Step II RC2v1_Fwd	5′-gggaagtcggggcccgggccccgcccccggcccgttcctcgttagcatgcatatggatcctatggcgcggttctttgaaagcagtcgag-3′
Step II RC2_Rev	5′-gccggggcgctgcttgttctccgacgccatcgccgatgcggggcgatcctgccgccgccgccacctct-3′
Step I gD_Fwd	5′-ttgtcgtcatagtgggcctccatggggtccgcggcaaatatgccttggcgacccctatttgtttatttttct-3′
Step I gD _Rev	5′-atcgggaggctggggggctggaacgggtccggtaggcccgcctggatgtgttatttgttaactgttaattgtc-3′
Step II gD _Fwd	5′-ttgtcgtcatagtgggcctccatggggtccgcggcaaatatgccttggcggagaattccgatatccagatgacccagtccc-3′
Step II gD _Rev	5′-atcgggaggctggggggctggaacgggtccggtaggcccgcctggatgtgggatccaccggaaccagagc-3′
Taqman DNApolFw	5′-catcaccgacccggagagggac-3′
Taqan DNApolRev	5′-gggccaggcgcttgttggtgta-3′
Taqman Probe	FAM-ccgccgaactgagcagacacccgcgc-Tamra

### Viral rescue, production and titration and RealTime PCR analysis

For viral rescue, 1E + 05 SKOV3 cells cultured in 24-well plates were transfected with 250 ng of BAC-HSVs DNA with Lipofectamine Transfection Reagent (Life Technologies, Inc.) and grown up until full cytopathic effect (CPE) was reached. Starting from this step, viral particles were used to infect SKOV3 in a scale-up process to get appropriate quantities of viruses. To titrate infectious viral particles, plaque assays were performed. Briefly, on day -1, 2.5E + 05 SKOV3 cells were seeded in a 12-well plates; at day 0, viral sample were diluted, from 1:10 to 1:10E + 09, in low serum RPMI medium in a final volume of 350 µL, and incubated with SKOV3 by gently shaking 90′ at 37 °C. The media were replaced with 1 ml of low serum RPMI medium, and cells were cultured in a humidified atmosphere containing 5% CO_2_ at 37 °C. 120 hours later, cells were fixed with 100% ethanol for 10′, stained with 10% GIEMSA for 15′ and extensively washed with distilled water; plaques were counted to analyse infectious titer. To analyse the viral replication, the viral genome copies were titrated by TaqMan RealTime PCR (Taqman universal PCR mastermix, Applied Biosystems) from cell lysates. Briefly, viral samples were diluted in liquid formulation A195 and treated with RNase-free, DNase I recombinant enzyme (Roche) for 30′ at 37 °C to eliminate envelope-free viral DNA. The DNase I was inactivated with 25 mM (final concentration) EDTA for 20′ at 80 °C. Viral DNA was thus extracted from enveloped HSV-1 particles by SDS 0.1% (w/v, final concentration) and 100 µg proteinase K (Roche) for 1 hour at 56 °C. The extracted viral particles were diluted 1:10, 1:100 and 1:1000 and analysed by TaqMan RealTime PCR according to the manufacturer’s recommendations (oligoes and probe in Table 1). R-LM55 and R-LM113 viruses were produced by infection of SKOV3 cells with 0.1 plaque-forming unit (PFU)/cells.

### *In vivo* studies

Female heterozygous C57 B6.Cg-Pds5b<Tg(Wap-ERBB2)229Wzw > /J mice were used for *in vivo* studies. Mice were implanted subcutaneously on the right flank with 5 × 10^5 LLC-ERBB2 cells. Ten days after challenge, mice bearing established tumours were randomized according to tumour size, and 5E + 07 viral PFU were injected intra tumour in combination with intra-peritoneally treatment with 200 μg α-mPD-1 (BioXcell, clone RMP114) according to the following schedule: oncolytic R-LM113 or SurE_oHSV on day 0, 2, 4, 7; aPD-1 on day 0, 3, 7, 10, 14, 17. An untreated and a α-mPD-1 treated group were used as control. The growth of tumours was measured by caliper every 3 or 4 days using the formula (LxW^2^)/2^31^. Animals were sacrificed as soon as signs of distress or a tumour volume above 1500 mm^3^ occurred. On day 44, cured mice received a second subcutaneous challenge of tumour cells on the left flank. The experimental procedures were approved by the Italian Ministry of Health (Authorizations 213/2016 PR) and have been done in accordance with the applicable Italian laws (D.L.vo 26/14 and following amendments), the Institutional Animal Care and ethic Committee of CEINGE and Allevamenti Plaisant SRL.

### Statistical analysis

For statistical analysis, T-Test or Two-way ANOVA were performed by GraphPad Prism and reported according to the following code <0.05 *; <0.005 **; <0.0005 ***; <0.00005 ****. For *in vivo* studies, statistical significance was calculated by mid-P and Fisher’s exact test.

## Results

### Selection of the tumour-restricted Survivin/*BIRC5* promoter for engineering of a replication conditional herpesvirus

**Figure 1:**
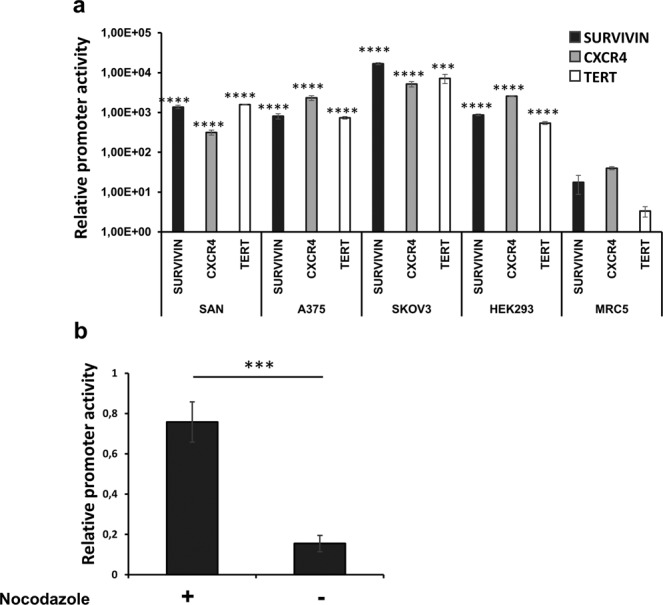
Evaluation of promoter selectivity for tumour cell lines. The activity of the selected gene promoters was assessed by SEAP reporter assays. (**a**) The activity of Survivin/*BIRC5*, CXCR4 and TERT promoters was assessed in tumour cells (SAN, A375, SKOV3, HEK293) and in normal lung fibroblasts (MRC5). The chart reports the relative strength of each promoter in the indicated cells, normalized to the activity of the housekeeping CMV enhancer/promoter construct. Statistical significance compares the promoter strength in normal MRC5 to corresponding tumour cell line. The asterisks indicate the P values: ***P < 0.0005; ****P < 0.00005 in the comparisons to the reporter activities shown by MRC5 cells. (**b**) SKOV3 cells were enriched in the G2/M phase of the cell cycle with 0.1 µg/ml of nocodazole in DMSO for 12 hours; Survivin/BIRC5 promoter activity of G2/M synchronized cells was compared to unsynchronized, DMSO-treated SKOV3 cultures. The asterisks indicate the P value: ***P < 0.0005.

To identify a suitable tumour-specific promoter for the generation of an advantageous replication conditional HSV-1, we explored the *CXCR4*, Survivin/*BIRC5* and *TERT* genes, known for their high level of expression in tumours^32–37^. In order to assess the extent of their cancer-restricted expression, we performed a Pan-Cancer analysis by the Cancer Genome Atlas (TCGA) Firebrowse Data portal (Institute TCGA Genome Data Analysis Center (2016): Firehose stddata__2017.9.24 run. Broad Institute of MIT and Harvard) and verified that these genes are indeed strongly expressed in a wide set of human tumours. Compared to Survivin/*BIRC5* and *TERT*, the expression of *CXCR4* resulted not adequately selective for tumour samples *versus* healthy tissues. In contrast, Survivin/*BIRC5* and *TERT* genes showed a prominent tumour-enriched expression. The trend of relative expression (cancer vs. healthy tissues) was indeed more favourable for Survivin/*BIRC5* than for *TERT* (Supplementary Fig. 1). Next, we engaged promoter searches for each of the three genes, to find the gene regulatory regions, able to recapitulate those features. By combining literature reports with bioinformatic tools of regulatory elements prediction (PROMO) and Encyclopedia of DNA Elements (ENCODE), we identified the putative promoter sequences for the three analysed genes in the regions −260 to −1 for Survivin/*BIRC5*, −400 to −24 for *TERT*, and −300 to −1 for *CXCR4* (positions are relative to the corresponding translation initiation start sites)^38–44^. Based on this information, we generated reporter gene constructs by cloning the selected promoters upstream of the secreted alkaline phosphatase cDNA (SEAP). The reporter vectors were transfected into 4 human transformed cell lines, SAN and A375 (malignant melanoma), SKOV3 (ovarian adenocarcinoma) and HEK293 (transformed human embryo kidney), as well as in human MRC5 cells (normal lung fibroblasts)^45^. Figure 1a shows the results of reporter activity for each promoter, normalized to the activity of the ubiquitous cytomegalovirus enhancer/promoter construct. All the tumour promoters were strongly active in the cancer cell lines, from 1 to 3 orders of magnitude, compared to the low activity in MRC5 normal fibroblasts. Based on the lower strength enrichments in tumour *vs*. normal tissues (Supplementary Fig. 1) and on reporter analysis (Fig. 1a), we excluded *CXCR4*. In contrast, *TERT* and Survivin/*BIRC5* promoters appeared more selective for tumour tissue expression and showed the best relative increases in reporter activity in the tumour cell lines. Finally, based on RSEM values, we focused our subsequent efforts on Survivin/*BIRC5* promoter to address a more sustained expression of viral gene of interest (Supplementary Fig. 1). Survivin/*BIRC5* promoter was also the most active promoter in the SKOV3 cell line (Fig. 1a), a well characterized and optimal system for production of ERBB2-retargeted HSV-1 vectors^14^.

Since Survivin/*BIRC5* is highly transcribed at the G2 phase of the cell cycle^46^, we aimed to confirm that the selected promoter region (−260/−1) was actually recapitulating its physiological responsiveness; so, we used nocodazole to enrich transfected cell populations in the G2/M phase of the cell cycle. Accordingly, the reporter assay in Fig. 1b shows that the activity of Survivin/*BIRC5* promoter in nocodazole-treated SKOV3 cells was actually increased, in comparison to untreated cells. Thus, the −260/−1 region of the Survivin/*BIRC5* promoter fully recapitulates the behaviour of the encoded gene in cancer cell lines.

### The Survivin_oHSV replication conditional virus shows a tumour cell-restricted lytic potential

**Figure 2:**
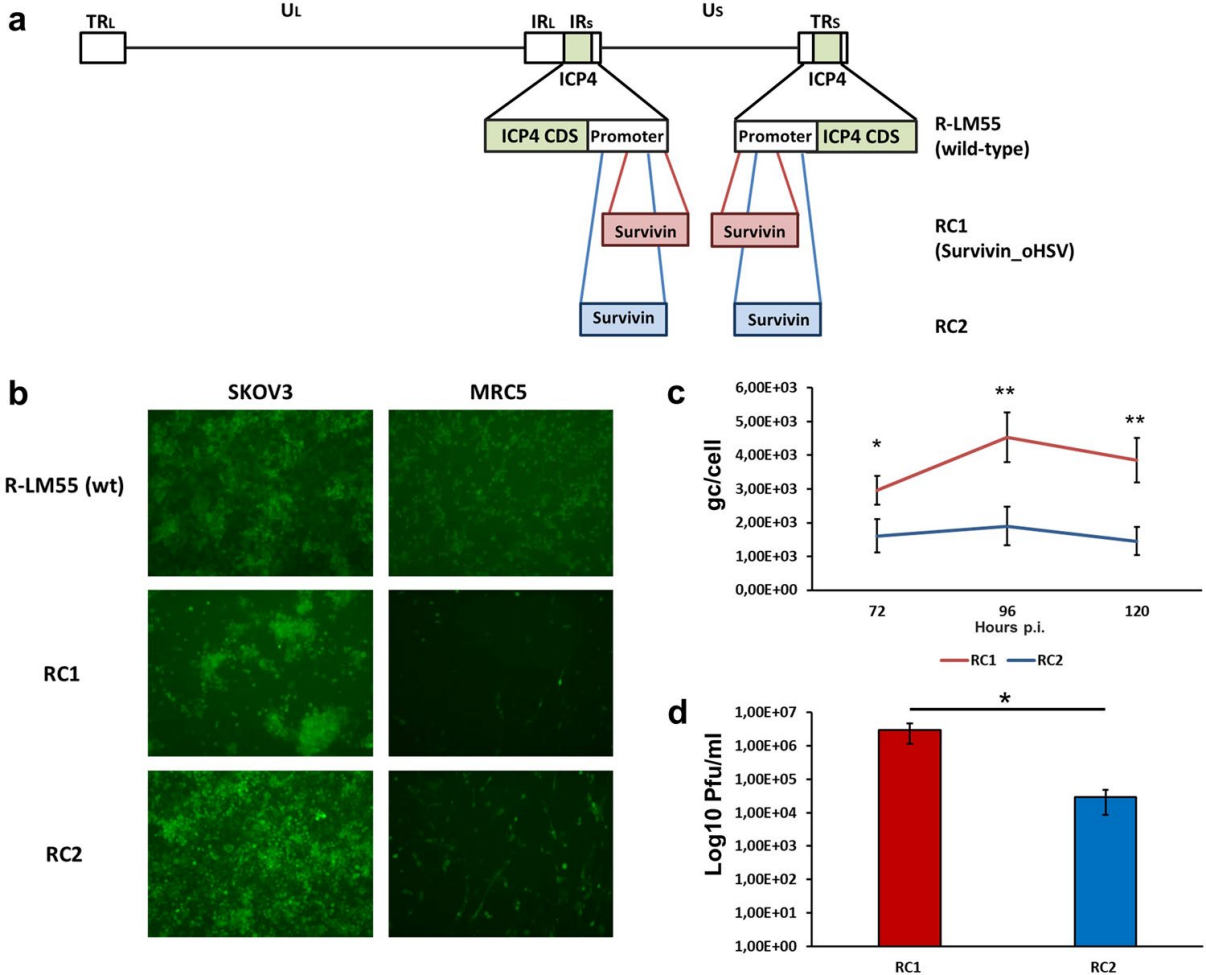
The Survivin/BIRC5 promoter confers conditional replicative potential to HSV-1 in SKOV3 cells. (**a**) The cartoon shows the regions of the endogenous ICP4 promoters from positions −650/−300 (layout RC1) and −600/−20 (layout RC2), replaced by the Survivin/BIRC5 promoter. (**b**) The spreading ability of wild-type R-LM55, RC1 and RC2 in SKOV3 (tumour) and MRC5 (normal) infected cells (0.1 PFU/cell) was evaluated by fluorescence microscopy (left and right columns, respectively). (**c**) Evaluation of the replicative potential of RC1 and RC2 viruses in SKOV3 cells infected with 0.1 PFU/cell. The qPCR-TaqMan analysis revealed the average genome copies per cell (gc/cell) produced by both viruses at the indicated timepoints. (**d**) Evaluation of the viral titers obtained in SKOV3 cells with RC1 and RC2 viruses (0.1 PFU/cell) by plaque assays. The asterisks in the Panels c and d indicate the P values: *P < 0.05; **P < 0.005.

We used the Survivin/*BIRC5* promoter to drive the transcription of alpha4 gene, encoding for the essential ICP4 viral protein of HSV-1, to evaluate whether the replacement of the endogenous promoter with the −260/−1 fragment of Survivin/*BIRC5* would restrict the replication of the novel herpesvirus to cancer cell lines. Starting from the wild-type HSV-1 strain F virus containing BAC sequence (hereinafter referred to as R-LM55), we generated the recombinant virus through the recombineering cloning approach. The *ICP4* gene is present in two copies in the HSV-1 genome so, both copies of the natural viral promoter were replaced with Survivin/*BIRC5* sequences in two different deletion/insertion sites, −650/−300 for construct RC1^20^ and −600/−20 for construct RC2^22^, relative to each of the translation start sites of the two copies of the alpha4 genes (see Fig. 2a for a schematic view). To characterize the corresponding viruses, tumour SKOV3 and non-tumour MRC5 cells were infected with the wild-type (R-LM55) and with the RC1 and RC2 viruses at multiplicity of infection (MOI) of 0.1 PFU/cell. Viral infection and propagation were monitored via fluorescence detection, thanks to the BAC-encoded enhanced green fluorescent protein (eGFP) gene. Monitoring of the infections after 72 h (Fig. 2b) revealed that both RC1 and RC2 viruses showed poor replication and spread in MRC5 cells, compared to the wild-type counterpart, R-LM55. The presence of isolated, green MRC5 cells upon infection with both RC1 and RC2 suggested that the replication conditional viruses were able to enter normal cells, and were not able to replicate efficiently in the non-transformed cellular background. In contrast, conditional recombinants exhibited robust spread in SKOV3 tumour cells (Fig. 2b). In order to select the most advantageous virus in terms of viral growth, we performed quantitative assays of viral replication and production of infectious viral particles for RC1 and RC2 viruses. SKOV3 cells were indeed infected with 0.1 PFU/cell with each virus. The quantitative TaqMan real-time PCR assays revealed that at all the analysed time points (72, 96, 120 hours post-infection) the genome copies produced per cell by RC1 were at least double, compared to RC2 (Fig. 2c). The viral yield obtained from infected SKOV3 cells showed that the RC1 virus was able to produce infectious virus particles much more efficiently (2 orders of magnitude higher) than to RC2 virus (Fig. 2d). Given the higher replicative potential and the more efficient viral production exhibited by the RC1, this latter was selected for further characterizations, and named Survivin_oHSV.

**Figure 3:**
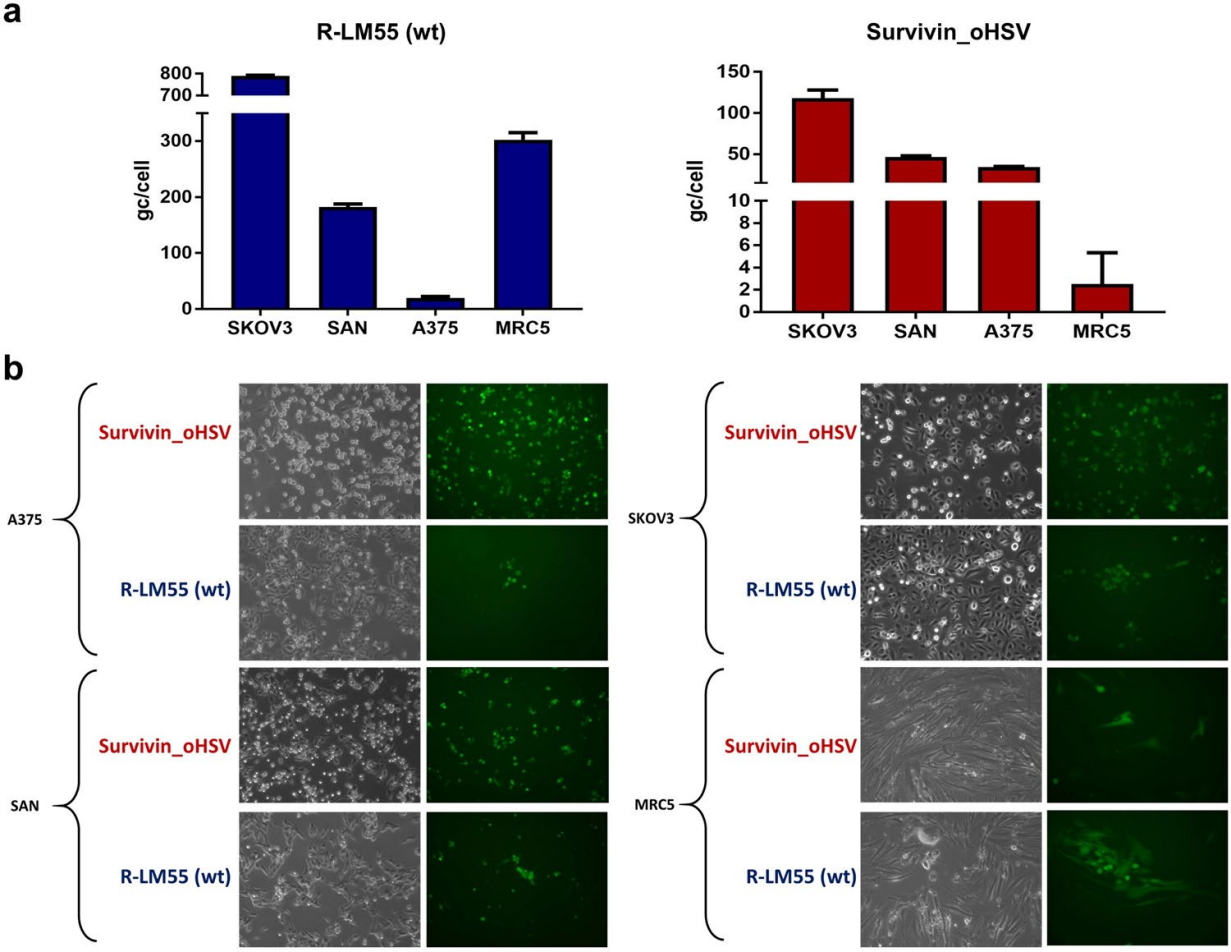
Evaluation of viral replication and cytopathic effect of Survivin_oHSV in tumour and normal cell lines. (**a**) Replicative potential of the wild-type R-LM55 and of Survivin_oHSV were evaluated in the survivin positive tumour cell lines (SKOV3, SAN and A375), and in normal MRC5 cells. Cells were infected at MOI 0.1 (PFU/cell), and the genome copies were determined 24 h post infection. All the differences in viral genome copies replication are statistically significant (≤0.01). (**b**) The panels show the viral replication 24 h post infection at 0.1 multiplicity of infection with the RC Survivin_oHSV and wt R-LM55 viruses. The viral spread is evaluable as eGFP positive cells under fluorescence microscopy; the cytopathic effect is evident as round and dethatched cells by phase-contrast microscopy.

Next, we analysed the oncolytic potential of Survivin_oHSV in additional cancer cell lines. The data reported in Fig. 3a show that viral replication of Survivin_oHSV was, in general, less efficient, compared to the wild-type virus R-LM55; however, Survivin_oHSV replicated to a higher extent in tumour cell lines (SKOV3, SAN and A375) compared to MRC5, with the highest yield in SKOV3 cells, in agreement with the reporter assay data for the Survivin/*BIRC5* promoter (Fig. 1a). Non-tumour MRC5 cells were confirmed refractory to sustain viral replication of Survivin_oHSV, while sustained effective R-LM55 wild-type virus replication. Interestingly, Survivin_oHSV replicated to similar yield as wild-type R-LM55 in A375 cells, indicating an optimal tumour/virus combination dependent mechanism (Fig. 3a).

The replicative competence of the Survivin_oHSV in the selected cancer cell lines *versus* normal cells was in agreement with the data shown in Fig. 3b, where the viral spread (eGFP expression) and the induced cytopathic effect (round, detaching cells) were documented. In MRC5 fibroblasts, the observed “single cell” green phenotype suggests that the Survivin_oHSV viruses entered normal cells, but failed to replicate efficiently in Survivin/*BIRC5* promoter non-competent background. On the contrary, the spread of the wild-type R-LM55 virus in normal MRC5 cells is unrestricted, and is similar to that observed in tumour cell lines (Fig. 3b). Despite the tumour-preferential replication of Survivin_oHSV was confirmed in different experimental conditions, a residual replication and toxicity of Survivin_oHSV is observable in non-tumour MRC5 cells infected at high MOI at late time point of infection (Supplementary Figs. 2 and 3).

### ERBB2 retargeting confers improved tumour cell selectivity to Survivin_oHSV virus

**Figure 4:**
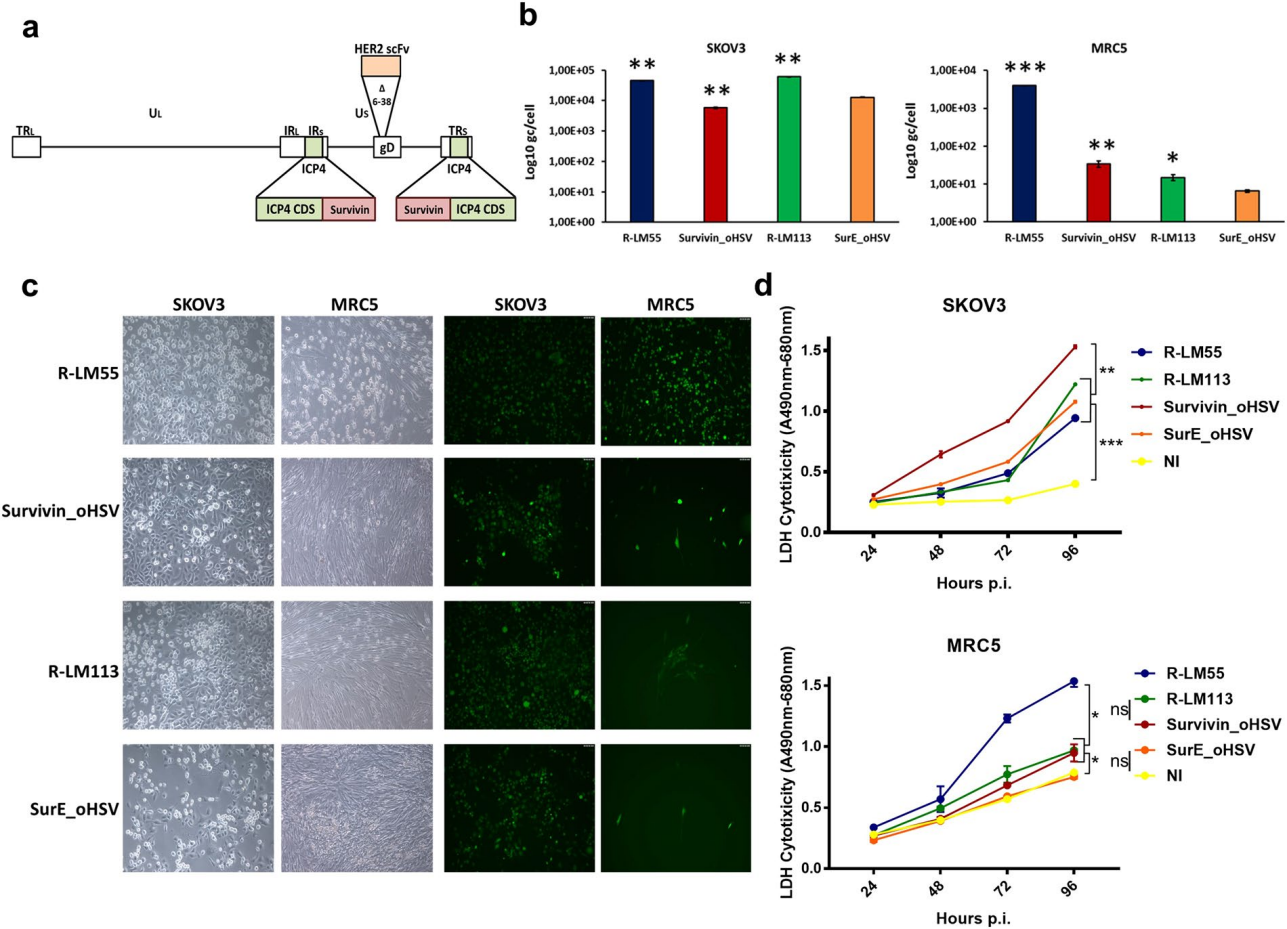
Schematic representation of the genome of the replication conditional, ERBB2-retargeted SurE_oHSV virus. (**a**) The region of the gD glycoprotein (6–38 aa) in the Survivin_oHSV backbone was substituted by a ERBB2 scFv sequence for general detargeting from cellular receptors mediating viral entry and retargeting to ERBB2^+^ cells. The new oncolytic virus was named SurE_oHSV (SurvivinErbb2_oncolyticHSV). (**b**) Replicative potential and spread (**c**) of double regulated SurE_oHSV were evaluated in comparison to single regulated viruses (Survivin_oHSV and R-LM113) and wild type R-LM55 virus in tumour SKOV3 and normal MRC5 cells infected with 0.03 PFU/cell. Viral genome copies were titrated 48 h post infection. Statistical significance was calculated comparing SurE_oHSV to all the other viruses (**d**). Cytotoxicity induced by wild type, single and double regulated viruses in normal MRC5 and tumour SKOV3 cells was evaluated according to Lactic Acid Dehydrogenase (LDH) release in cell supernatant in time course of infection from 24 to 96 hours post infection with 0.1 PFU/cell. The asterisks in the Panels b and d indicate the P values: *P < 0.05; **P < 0.005; ***P < 0.0005.

Analysis of Survivin_oHSV replication showed that the *ICP4* conditioning by Survivin/*BIRC5* promoter mediated up to 3 orders of magnitude of selectivity for cancer cells as compared to non-tumour MRC5 fibroblasts. The relatively low replication of Survivin_oHSV in normal cells, was described earlier, and is presumably due to residual promoter leakiness (Supplementary Fig. 2)^25^. We reasoned that adding a retargeting feature to the replication conditional virus would decrease the off-tumour replication and thus improve the selectivity of Survivin_oHSV for tumour cells, and its safety in clinical settings. To this end, we took advantage of engineered viral glycoprotein gD fused to an antibody fragment targeting ERBB2^14,47^. This additional feature abrogates the virus ability to enter cells through the natural receptors HVEM/Nectin-1, and enables the specific infection of cells overexpressing ERBB2 in addition to the conditional replication through Survivin/*BIRC5* promoter. Figure 4a shows a schematic view of the genome organization of the recombinant virus, named SurE_oHSV (Survivin-ERBB2 oncolytic *HerpesSimplex* Virus-1). To evaluate the tumour-restricted replication of the novel virus, SKOV3 (ERBB2+, Survivin promoter permissive) and MRC5 (ERBB2+/−, Survivin promoter non-permissive) cells were infected with one of the following viruses: the wild-type R-LM55, the replication conditional Survivin_oHSV, the ERBB2-retargeted R-LM113^14^ and the combined SurE_oHSV viruses (see Table 2 for description of the viruses). The ERBB2-retargeted virus, R-LM113, and the SurE_oHSV share the entry mechanism, but differ in the promoter driving *ICP4* expression. As shown in the Fig. 4b, SurE_oHSV exhibited a decreased yield in SKOV3 cells as compared to R-LM113; this is in agreement with the decreased replication rate of parental Survivin_oHSV virus, in the comparison with the wild-type R-LM55 (Fig. 2b). SurE_oHSV also showed a decreased replication in normal MRC5 cells, as compared to R-LM113 (Fig. 4b). However, both SurE_oHSV and R-LM113 displayed similar cell to cell spread and cytopathic effects (Fig. 4c), as well as cytotoxic properties (Fig. 4d) in SKOV3 cells. In contrast, MRC5 cells were more refractory to SurE_oHSV spread and cytopathic effects (Fig. 4c), as well as to cytotoxicity (Fig. 4d), in comparison to R-LM113.

**Table 2:** Summary of the features of the HSV-1 vectors used in this study.

Virus	*ICP4* promoter	Entry receptors	Reference
R-LM55 (wild-type)	Wild-type	HVEM/Nectin-1	^14^
Survivin_oHSV	Survivin/*BIRC5*	HVEM/Nectin-1	Current study
R-LM113	Wild-type	ERBB2	^14^
SurE_oHSV	Survivin/*BIRC5*	ERBB2	Current study

The double retargeted SurE_oHSV and the parental Survivin_oHSV share the promoters driving *ICP4* transcription, while they differ for the entry mechanism (Table 2). Interestingly, SurE_oHSV gained improved replication over the parental Survivin_oHSV in SKOV3 tumour cells (Fig. 4b). Correspondingly, SurE_oHSV also exerted a higher cytopathic effect against SKOV3 cells assessable as round detached cells (Fig. 4c) which, however, did not translate into an improved cytotoxic effect measured as extracellular Lactic Acid Dehydrogenase (LDH) release (Fig. 4d). This discrepancy could presumably be due to different replication kinetics resulting in the lack of cell substrates at late time points of infection for the more virulent viruses (Fig. 4c and Supplementary Fig. 4). A detailed representation of the cytopathic effect and spreading ability exerted by the SurE_oHSV virus in tumour SKOV3 cells is shown in the Supplementary Fig. 4. Taking into account the effects exerted by the double retargeted and the parental Survivin_oHSV in normal MRC5 cells, SurE_oHSV behaved more safely than Survivin_oHSV and R-LM113 for each of the features described in Fig. 4, i.e., decreased replication (Fig. 4b), cytopathic effect, spreading (Fig. 4c), and cytotoxicity (Fig. 4d).

To further confirm that ERBB2 retargeting actually implements the tumour selectivity of replication conditional Survivin_oHSV, we took advantage of the syngeneic system offered by wild-type murine LLC1 cell line and its transgenic human ERBB2-expressing counterpart^27^. As depicted in Supplementary Fig. 5, the ERBB2 expressing LLC1 cells were infected equally well through both the non-restricted (R-LM55 and Survivin_oHSV) and the target-mediated (SurE_oHSV) entry mechanisms. Contrariwise, in the absence of human ERBB2 expression, the infection of wild-type LLC1 cells by SurE_oHSV was completely abrogated, while the same cells were still permissive to R-LM55 and Survivin_oHSV infection. Thus, the double retargeted SurE_oHSV shows a good oncolytic activity and improved safety features in non-transformed cells, which render it a suitable candidate for preclinical evaluations.

### The replication conditional, ERBB2 retargeted SurE_oHSV in combination with PD-1 blockade maintains the efficacy of the parental ERBB2-retargeted virus *in vivo*

**Figure 5:**
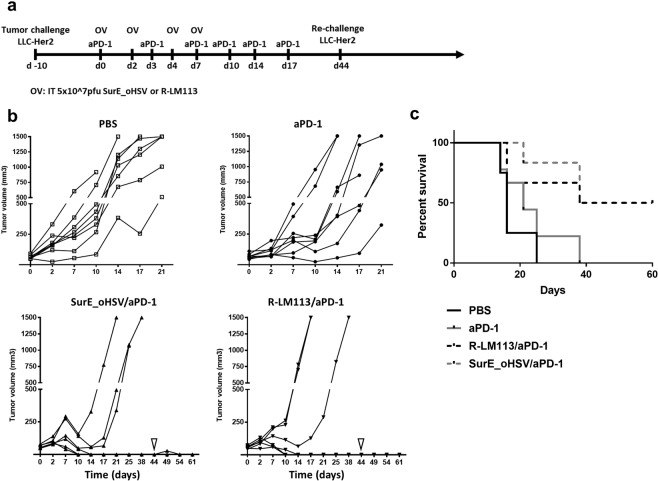
Efficacy of SurE_oHSV in combination to PD-1 immune checkpoint blockade in mouse syngeneic established tumour model. (**a**) Schedule of the treatment. (**b**) Efficacy of oncolytic virus (SurE_oHSV or R-LM113) and anti PD-1 antibody combination. The four experimental groups were composed as follow: untreated (white squares; N = 8 mice), aPD-1(black circles; N = 8 mice), SurE_oHSV/aPD-1 (up-pointing black triangles; N = 6 mice), R_LM113/aPD-1 (down-pointing black triangles; N = 6 mice). Oncolytic viruses were administered intratumourally (0.5 × 10^8^ PFU/injection) on days 0, 2, 4, 7; anti PD-1 antibody was systemically administered on days 0, 3, 7, 10, 14, 17 by intraperitoneal injections. Cured mice received a second tumour challenge on day 44 (down-pointing white triangles). Fisher test aPD-1 to SurE_oHSV or R-LM113 viruses = 0.05. (**c**) Kaplan-Meier survival analysis of the experiment outlined in Fig. 5a.

The *in vitro* characterization of SurE_oHSV virus highlights a strong potential in exploiting its effectiveness in preclinical settings of cancer therapy. Accordingly, we evaluated anti-tumour efficacy in immune-competent transgenic mice, tolerant to human ERBB2, harbouring ectopic, established tumours based on ERBB2-positive LLC1 murine cancer cells^27^. The LLC1 established tumours raised in syngeneic C57B6 mice are poorly responsive to oncolytic virotherapy and to immune checkpoint blockade monotherapy^27,48,49^. Mice injected with ERBB2-positive LLC1 cancer cells, upon appearance of tumours > =100 mm^3^ in volume were randomized and subjected to *placebo*, or to anti-PD1 antibody (days 0, 3, 7, 10, 14, 17 from appearance of tumour), or to combined anti-PD1 treatments (schedule as above) with either R-LM113 or SurE_oHSV viruses (0.5 × 10^8^ PFU at days 0, 2, 4, 7) (Fig. 5a). The Fig. 5b reports the tumour growths. Responders were defined as complete, upon absence of tumour relapse after 40 days. The charts reporting the tumour volumes highlight a potent therapeutic efficacy in mice exposed to combination therapy of aPD-1 with either R-LM113 (50% complete responders) or SurE_oHSV (50% complete responders). Moreover, all the cured mice were re-challenged in the opposite flank at day 44 and resulted resistant to ERBB2-positive LLC1 cells (Fig. 5). All the cured mice resulted long-term survivors with absence of disease-recurrence even after tumour re-challenge (Fig. 5c). Altogether, these data show that R-LM113 and SurE_oHSV oncolytic viruses actually synergize with PD-1 immune checkpoint blockade, elicit similar efficacy in the preclinical setting of LLC1 cells established tumours and drive an abscopal effect after tumour re-challenge in cured mice.

## Discussion

Since T-VEC approval in 2015 for the treatment of advanced-stage unresectable melanoma, several oncolytic viruses have successfully proved their safety and efficacy in different preclinical models and are currently under clinical evaluations^29,30,50^. Nevertheless, emerging evidences suggest that there is still room for improvements of oncolytic virotherapy through combination therapies (e.g. immune checkpoint blockade, immunomodulatory payloads) and use of more virulent isolates or non-attenuating engineering strategies^51^. The HSV-1 based oncolytic platform possesses several relevant features: (i) the clinical validation and the possibility to counteract adverse effects by antiviral drugs^52^; (ii) a large and easy-to-manipulate genome that allows to host payloads of interest; (iii) genetically editable envelope glycoproteins to target cancer cells; (iv) the proven ability to act as a cancer vaccine conferring immunological memory against treated tumours^27,53^. To date, attenuation by gene-inactivating deletions has been widely applied to HSV-1, but this strategy can limit the efficacy of oHSVs since it can compromise the virulence also in tumour cells^54^, and affect the viral ability to escape host’s antiviral immune pathways (e.g. cGAS/STING axis)^55,56^.

With the aim to improve our current HSV-1 oncolytic platform, we generated and characterized a virulent oncolytic herpesvirus, SurE_oHSV, bearing two independent features i.e., replication conditioning by Survivin/*BIRC5* promoter control of the viral *ICP4* gene, and a combination of de-targeting (from common entry receptors) and re-targeting (guided by ERBB2 receptor expression) approach^14^. Retargeting by engineered glycoprotein D has been already demonstrated to ensure de-targeting from natural receptors of HSV-1 and new tropism for selected tumour-associated receptors^15^. Unfortunately, tumour-associated receptors do not clearly differentiate tumour from non-tumour cells, making retargeting approaches potentially not exempt from on-target, off-tumour toxicity, similarly to CAR-T cells^57–60^. Thus, adding additional features to a retargeted virus, such as replicative conditioning, may result in the generation of non-attenuated viral vectors for efficient lysis of cancer cells, tumour debulking and consequent elicitation of immunogenic cell death.

For replicative conditioning, the fragment representing the Survivin/*BIRC5* promoter was identified and proven to recapitulate the optimal expression features of the reference cellular gene in terms of transcription strength, tumour selectivity and cell cycle regulation. Its insertion into two alternative sites of the *ICP4* gene pair identified a preferential site; both substituted regions encompass the endogenous core promoter and transcriptional start site of *ICP4*^61^ and lead to Survivin/*BIRC5*-dependent transcription of alpha4 gene and tumour-preferential replication. Despite both recombinant viruses (RC1, RC2) showed tumour-enriched replication, RC1 resulted more efficient than RC2 in replication and viral particle formation. Since the main difference between RC1 and RC2 affected the 5′-untranslated region of *ICP4*, being maintained in RC1 while abolished in RC2, we can speculate that, as reported for additional herpetic genes^62^, this region could have a functional role in *ICP4* gene expression. The RC1 (Survivin_oHSV) herpesvirus, showing proper replicative potential and efficient production of infective viral particles was accordingly employed for introduction of the ERBB2 receptor targeting strategy, which allowed the generation of the double regulated HSV-1 oncolytic vector, SurE_oHSV.

The SurE_oHSV virus displayed improved safety features, compared to the wild-type HSV-1, or to the viruses holding either the replication conditioning feature (Survivin_oHSV), or the ERBB2 retargeting capability (R-LM113). In fact, SurE_oHSV was characterized by a limited ability to infect and propagate into the normal cellular background of human fibroblasts, while keeping a suitable replicative and cytotoxic potential in tumour cell lines.

To determine whether the oncolytic activity was actually maintained *in vivo*, we evaluated the efficacy of the SurE_oHSV virus versus the retargeted-only R-LM113 in a highly aggressive preclinical setting based on immunocompetent mice. Earlier *in vivo* studies demonstrated that the experimental setting based on established LLC1-ERBB2 derived tumours is actually resistant to retargeted oHSV^27^. We show here that LLC1-ERBB2 established tumour model is also resistant to immunotherapy (i.e. PD-1 checkpoint blockade), but it can become susceptible to treatment when retargeted oHSVs are administered in combination with immune checkpoint inhibitors or is potentiated by immunostimulatory cargoes (i.e. mIL-12) (D’Alise *et al*., submitted). Here, we show that the combined treatment with PD-1 immune checkpoint blockade and double regulated SurE_oHSV or single retargeted R-LM113 virus show an effective synergistic effect, leading to elicitation of a systemic anti-tumour immunity that remarks the opportunity to exploit oncolytic viruses to overcome the immunosuppressive strategies adopted by tumour cells. The comparable activity of SurE_oHSV and the single retargeted R-LM113 virus reveals that the added feature of replication conditioning is not detrimental for oncolytic and immunotherapeutic features of the ERBB2-retargeted virus. This information, combined to the improved off-target toxicity exerted by SurE_oHSV in normal cells, allows to predict that it may represent a valid and safer option in virotherapy with decreased off-tumour infection and replication in healthy tissues. This consideration will be further endorsed by future toxicity studies, for which the setup of an appropriate susceptible model is required. In fact, in the same human ERBB2 tolerant animal system used in this paper, the safety profile of retargeted virus R-LM113 was already very high, as mice tolerated systemic high doses of virus (2E + 09 PFU)^27^. Although this satisfactory safety is mediated by de-targeting from endogenous ligand of HSV-1, the expression of human ERBB2 under the control of whey acidic protein (WAP) promoter^63^ does not fully recapitulate the orthotopic expression of the target and the potential on-target, off tumour-related toxicity. This limitation makes further studies in dedicated animal models required to address how complementing re-targeting with the added feature of replication conditioning might actually improve the safety of the oncolytic HSV-1. Accordingly, our results open up the possibility to generate a next generation repertoire of oncolytic herpes viruses that combine retargeting and replication conditioning, aiming to use this clinically validated oncolytic platform for intravenous delivery.

## Supplementary information

Supplementary Figured_revised.
