# Supplementary material for: Replicative conditioning of Herpes simplex type 1 virus by Survivin promoter, combined to ERBB2 retargeting, improves tumour cell-restricted oncolysis
Source: Sci Rep. 2020 Mar 9;10:4307. doi: 10.1038/s41598-020-61275-w (PMC7062820; doi:10.1038/s41598-020-61275-w)

Replicative conditioning of *Herpes simplex* type 1 virus by Survivin promoter, combined to ERBB2 retargeting, improves tumour cell-restricted oncolysis

Emanuele Sasso^1,2,3*^, Guendalina Froechlich^2^, Gabriella Cotugno^3^, Anna Morena D’Alise^3^, Chiara Gentile^1,2^, Veronica Bignone^3^, Maria De Lucia^3^, Biljana Petrovic^3^, Gabriella Campadelli-Fiume^4^, Elisa Scarselli^3^, Alfredo Nicosia^1,2,3^ and Nicola Zambrano^1,2^

^1^Dipartimento di Medicina Molecolare e Biotecnologie Mediche, Università degli Studi di Napoli Federico II, Via S. Pansini, 5 80131 Naples, Italy; ^2^CEINGE Biotecnologie Avanzate S.C.aR.L., Via G. Salvatore 486, 80145 Naples, Italy; ^3^Nouscom S.R.L., Via di Castel Romano 100, 00128 Rome, Italy; ^4^Department of Experimental, Diagnostic and Specialty Medicine, University of Bologna, Via San Giacomo 12, 40126 Bologna, Italy

*Corresponding Author

Emanuele Sasso [e.sasso@nouscom.com](mailto:e.sasso@nouscom.com)

Dipartimento di Medicina Molecolare e Biotecnologie Mediche, Università degli Studi di Napoli Federico II, Via S. Pansini, 5 80131 Naples, Italy; +39 0813737916

**Supplementary Figure 1. *In silico* evaluation of tumour-selective promoters by mRNASeq database analysis.** The panels show the analysis of mRNA expression from The Cancer Genome Atlas of the three target genes (BIRC5, hTERT, CXCR4). The boxes compare the expression of mRNAs in tumour tissues (red boxes) and into their normal counterparts (blue boxes). The missing reports are in grey. Abbreviations: ACC (Adrenocortical carcinoma), BLCA(Bladder Urothelial Carcinoma), BRCA(Breast invasive carcinoma), CESC(Cervical squamous cell carcinoma and endocervical adenocarcinoma), CHOL(Cholangiocarcinoma), COAD(Colon adenocarcinoma), COADREAD(Colorectal adenocarcinoma), DLBC(Lymphoid Neoplasm Diffuse Large B-cell Lymphoma), ESCA(Esophageal carcinoma), FPPP(FFPE Pilot Phase II), GBM(Glioblastoma multiforme), GBMLGG(Glioma), HNSC(Head and Neck squamous cell carcinoma), KICH(Kidney Chromophobe), KIPAN(Pan-kidney cohort(KICH+KIRC+KIRP)), KIRC(Kidney renal clear cell carcinoma), KIRP(Kidney renal papillary cell carcinoma), LAML(Acute Myeloid Leukemia), LGG(Brain Lower Grade Glioma), LIHC(Liver hepatocellular carcinoma), LUAD(Lung adenocarcinoma), LUSC(Lung squamous cell carcinoma), MESO(Mesothelioma), OV(Ovarian serous cystadenocarcinoma), PAAD(Pancreatic adenocarcinoma), PCPG(Pheochromocytoma and Paraganglioma), PRAD(Prostate adenocarcinoma), READ(Rectum adenocarcinoma), SARC(Sarcoma), SKCM(Skin Cutaneous Melanoma), STAD(Stomach adenocarcinoma), STES(Stomach and Esophageal carcinoma), TGCT(Testicular Germ Cell Tumours), THCA(Thyroid carcinoma), THYM(Thymoma), UCEC(Uterine Corpus Endometrial Carcinoma), UCS(Uterine Carcinosarcoma), UVM (Uveal Melanoma).

**Supplementary Figure 2.** **In vitro oncolytic activity of RC Survivin HSV and R-LM55.** To monitor the cytopathic effect in tumour (SKOV3) and normal (MRC5) cells, the two cell types were infected with Survivin_oHSV, or with the control, wild-type R-LM55 virus at different dosage (0.01, 0.02, 0.1, 0.5 PFU/cell) and for different time points post infection (up to 96h). The image shows two representative time points, 48h and 96h post-infection.

**Supplementary Figure 3. Evaluation of viral replication of Survivin_oHSV and wild-type R-LM55 in tumour and normal cell lines.** Replicative potential of the wild-type R-LM55 (blue lines) and of Survivin_oHSV (red lines) were evaluated in the survivin positive tumour cell lines (SKOV3 and A375), and in normal MRC5 cells. Cells were infected MOI of 0.03 PFU/cell, and the corresponding genome copies were determined at the indicated time points. ND: not detected.

**Supplementary Figure 4. Assessment SurE_oHSV spread over a range of MOI.**

To evaluate with better accuracy the toxicity of the SurE_oHSV virus, SKOV3 cells were infected over a wide range of MOIs, monitoring the cytopathic effect for up to 150 hours or to full cytopathic effect. The results showed that the SurE_oHSV was able to replicate, spread and induce cytopathic effects even at very low MOI (0.0005 PFU/cell).

**Supplementary Figure 5. Assessment of R-LM55, Survivin_oHSV and SurE_oHSV infection and spread in ERBB2 positive and negative LLC1 cells.** To assess the improved selectivity of SurE_oHSV compared to replication conditional Survivin_oHSV, LLC1 and human ERBB2 expressing counterpart were infected at MOI of 0.1 PFU/cell with wild type R-LM55, Survivin_oHSV and SurE_oHSV. The spread was monitored by eGFP expression up to 48h. The results showed that the SurE_oHSV was able to replicate and spread in LLC-HER2 cells but not LLC1 wild type cell line.


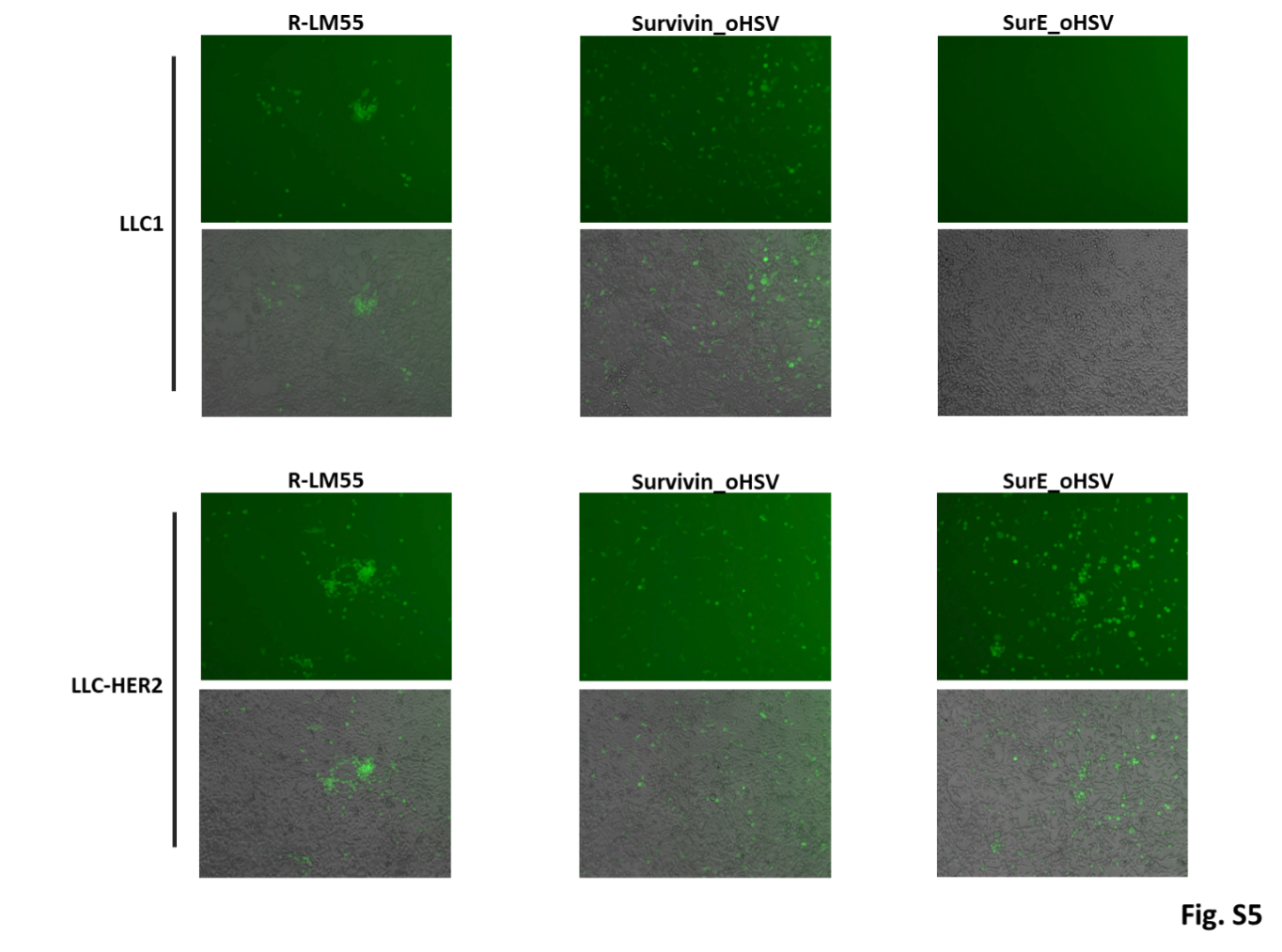

Supplement: Supplementary file 1 — Supplementary Figured_revised. [file 41598_2020_61275_MOESM1_ESM.docx]
